# Mycelium as a natural food fortificant: production, micronutrient enrichment, processing, and application in functional foods

**DOI:** 10.3389/fnut.2026.1890681

**Published:** 2026-08-13

**Authors:** Shubham Mandliya, Soubhagya Tripathy, Sourav Misra, Hari Niwas Mishra

**Affiliations:** 1 Agricultural and Food Engineering Department, Indian Institute of Technology Kharagpur, Kharagpur, India; 2 Department of Food and Nutritional Sciences, University of Reading, Reading, United Kingdom; 3 Division of Mechanical Processing, ICAR-National Institute of Natural Fibre Engineering and Technology, Kolkata, India

**Keywords:** fermentation, food fortificant, fortification, functional foods, micronutrients, mycelium

## Abstract

The vegetative body of filamentous mushrooms, known as mycelium, has attracted significant interest as a nutrient-rich, sustainable food ingredient that can help address micronutrient deficiencies and protein malnutrition worldwide. In contrast to synthetic fortificants, mycelium is a natural fortificant that bioaccumulates minerals (iron, zinc, selenium, etc.) and vitamins (vitamin B_12_ and vitamin D_2_) during fermentation, thus becoming a promising clean-label ingredient and fortificant. This review focuses on the nutritional (both macronutrient and micronutrient) profile of mycelium, including protein composition, minerals, vitamins, bioactives, etc. It also discusses different methods, i.e., submerged, solid-state, and precision fermentation for producing mycelial biomass. This study also explores micronutrient enrichment methods, such as biofortification, UV-assisted vitamin D_2_ enhancement, and encapsulation for mineral delivery. It discusses the application of mycelium in various functional foods, including meat substitutes, baked goods, dairy alternatives, and beverages. Additionally, the discussion covers sustainability and the environmental impact of mycelium, current challenges to its commercial use, and future developments. Mycelium offers a scalable, eco-friendly, and physiologically adaptable solution to hidden hunger, protein deficiency, and the drawbacks of traditional synthetic food fortification.

## 1 Introduction

**FIGURE 1:**
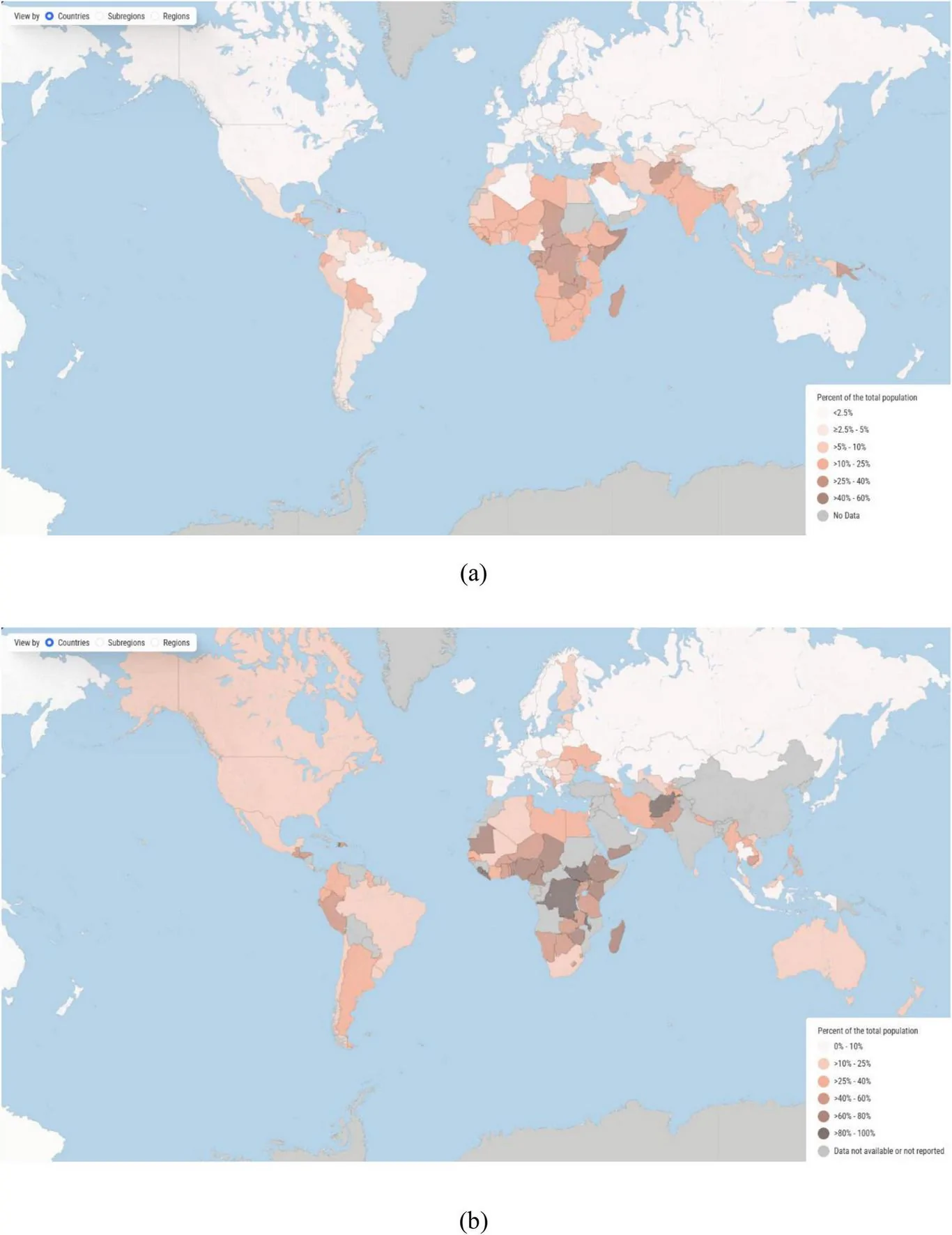
Prevalence of **(a)** undernourishment, and **(b)** moderate or severe food insecurity in the total population (percent) (3-year average – 2022–2024). Reproduced from FAO, licensed under CC BY 4.0.

Macronutrient and micronutrient deficiencies are fundamental issues for the global food system. These issues threaten public health, economic development, and even global progress. According to the recent reports by the Food and Agriculture Organization (FAO) in 2023, approximately 757 million people faced hunger, and by 2030, around 582 million people will suffer from malnutrition, with the majority residing in Africa (1). The prevalence of undernutrition and food insecurity is shown in Figure 1. One of the most common problems faced by the population is micronutrient deficiency, also termed as “hidden hunger” (2). Around 56% of preschool children (6–60 months age group) and 69% of women of reproductive age (15–49 age group) are deficient in at least one essential mineral (iron, zinc, selenium, etc.) or vitamin (B_9_, B_12_, D, etc.), according to estimates in a report published in The Lancet Global Health (3). To combat hidden hunger, one of the most common and widely used methods is to incorporate synthetic micronutrients into the formulation of food products. Some synthetic micronutrients include ferrous sulfate, ferric pyrophosphate (most commonly used in rice fortification), cyanocobalamin (vitamin B_12_), and ergothioneine (vitamin D_2_). However, these synthetic micronutrients face various challenges, including poor bioavailability, changes in sensory profile, regulatory guidelines, and consumer awareness and acceptance of these synthetic ingredients, making them not an ideal solution (2, 4–6). Iron bioavailability in fortified rice was low when ferrous sulfate was used as a synthetic additive; thus, ascorbic acid was added in many cases to increase bioavailability (5). Banik et al. (6) attempted to incorporate rice protein into rice flour and observed an increase in iron bioavailability from 52% to 78% when ferrous sulfate was added. Furthermore, their production includes energy-intensive chemical synthesis and generates tons of waste streams harmful to the environment, thereby increasing additional costs and also being ecologically harmful, making them not a first priority in today’s situation (7, 8). Together, these drawbacks support the investigation of clean-label, biologically derived substitutes for synthetic fortification that concurrently address production sustainability and nutritional adequacy within a single component platform.

**FIGURE 2:**
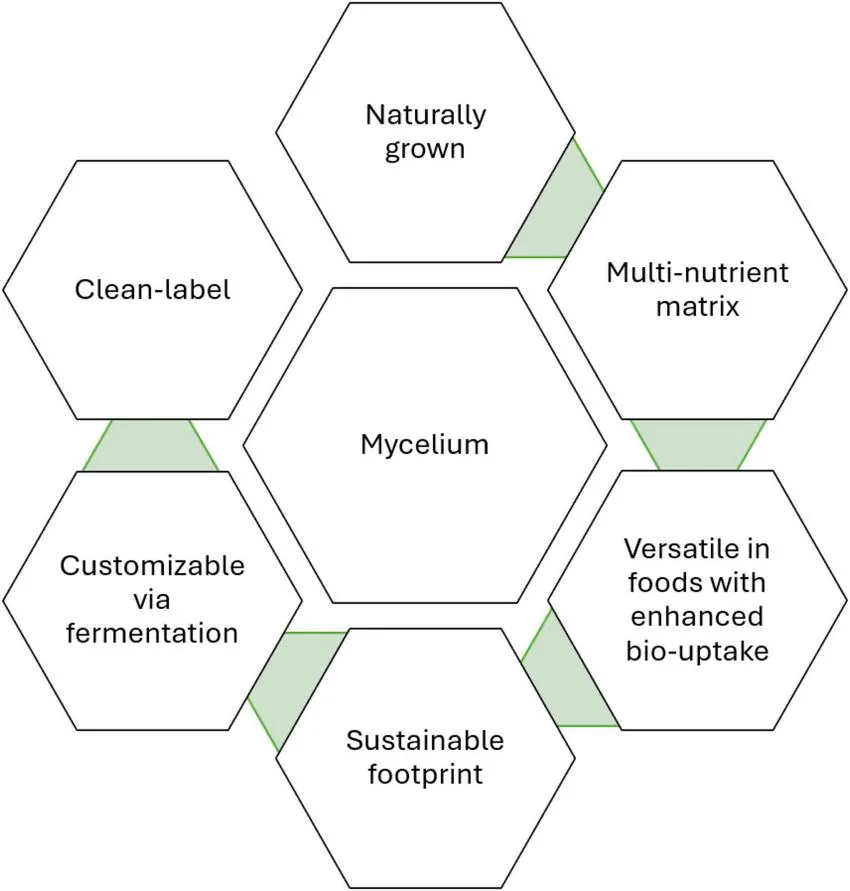
Key functional and strategic benefits of mycelium as a natural food fortificant.

Mycelium has recently emerged as a potential candidate for this challenge due to its growing conditions. In contrast to traditional plant and animal protein sources, mycelium has a unique advantage due to its versatile nutritional profile: high quality protein with all essential amino acids; a mineral-dense ash fraction that naturally accumulates iron, zinc, selenium, phosphorus, and potassium from the growth substrate through active biological uptake and biotransformation mechanisms; structural dietary fiber in the form of beta-glucans and chitin; low total fat content enriched in polyunsaturated fatty acids (9, 10). Mycelium is especially useful as a micronutrient delivery system for plant-and vegan-dietary patterns because it biosynthesizes several micronutrients, including ergocalciferol (Vitamin D_2_) upon UV irradiation, riboflavin, folate, and ergothioneine, whose plant-based analogs are mostly absent or poorly bioavailable (8, 11, 12). Mycelium requires 86% less water to produce a similar weight of protein as beef, and even produces 98% less greenhouse gas (GHG) emissions with 5.5 times less land, making it an environmentally friendly and future ingredient for food with an increasing population and limited resources (13, 14). Thus, mycelium is positioned as a clean-label, biologically programmed natural food fortificant that can accumulate and deliver micronutrients in organic, bioavailable forms without synthetic chemical intervention, and without the phytate-based antinutritional factors that restrict mineral bioavailability in plant proteins (15). Figure 2 summarizes these key functional and strategic benefits of mycelium as a natural food fortificant.

Mycelial biomass can be produced using different fermentation systems, each with unique nutritional and financial benefits. One system is submerged fermentation (SmF), which has been industrially used by Quorn for the production of *Fusarium venenatum* since 1985, under controlled fermentation conditions to achieve high yields and reproducible results (16). Another system is solid-state fermentation (SSF), which uses inexpensive agro-industrial residues, such as wheat bran, rice straw, sugarcane bagasse, and spent brewery grains, as both substrate and growth support, thereby producing high-density biomass with micronutrients, utilizing agricultural waste streams, aligning well with the concept of circular economy (17, 18). Recently, precision fermentation (PF) has gained attention for its ability to tailor mycelium production or even produce a specific component required using metabolic engineering, while reducing carbon emissions per unit of biomass produced (19, 20). All these fermentation systems offer a key benefit over traditional methods, which includes the ability to precisely customize nutrient profiles by adjusting process parameters. This precision enables targeted accumulation of specific micronutrients, such as selenium, zinc, iron, and vitamin D_2_, at levels suitable for food fortification (21, 22).

The nutritional quality of mycelium can be further improved after harvesting through downstream processing methods, such as drying, milling, and even UV irradiation, which converts ergosterol into vitamin D_2_ (12, 23, 24). Mycelium protein isolates can also be produced from mycelium, with good functional properties, such as emulsifying and foaming properties, along with essential amino acids and absorption capacity, and with a better secondary structure required for developing meat analogs (25–27). The microstructure of mycelium, with its chitin and β-glucans, makes it a suitable carrier for the encapsulation of micronutrients and bioactives, thereby enhancing the bioaccessibility of minerals in the gastrointestinal tract. It is also effective in extrusion, where it aligns into directional fibers resembling skeletal muscle architecture. Mycelium has been used in a wide variety of food products, such as a chicken substitute (28), low-moisture meat analog (24), chocolate cookies (29), functional beverage (30), bread (31), and tortilla (26) for protein supplementation, texture, or micronutrients such as iron or vitamin D_2_. These characteristics distinguish mycelium from standalone mineral supplements, which lack the food matrix.

Recent reviews have explored various aspects of mycelium as a food ingredient. Aguiar et al. (32) focused on processing methods, health benefits, and market trends of edible mushrooms and mycelium-derived products, emphasizing their applications rather than their nutrient-enrichment properties. Elhalis (33) discussed fungal mycelium for sustainable food solutions, highlighting the use of biomass and byproduct valorization from a circular bioeconomy perspective, but did not cover micronutrient enrichment or fortification techniques. Other reviews have narrowed their focus to mycelium’s bioactive compounds (34) or its potential as an alternative protein (35, 36), without integrating production, targeted micronutrient strategies, and functional food applications in a comprehensive fortification framework. Despite its enormous potential, mycelium production, micronutrient enrichment, processing methods, and applications in functional foods have not yet been thoroughly reviewed in the context of food fortification. Therefore, this review aims to provide a comprehensive overview by examining the nutritional profile of mycelium, its production methodologies, enrichment techniques, and its application in functional foods.

## 2 Nutrition profile of mycelium

**FIGURE 3:**
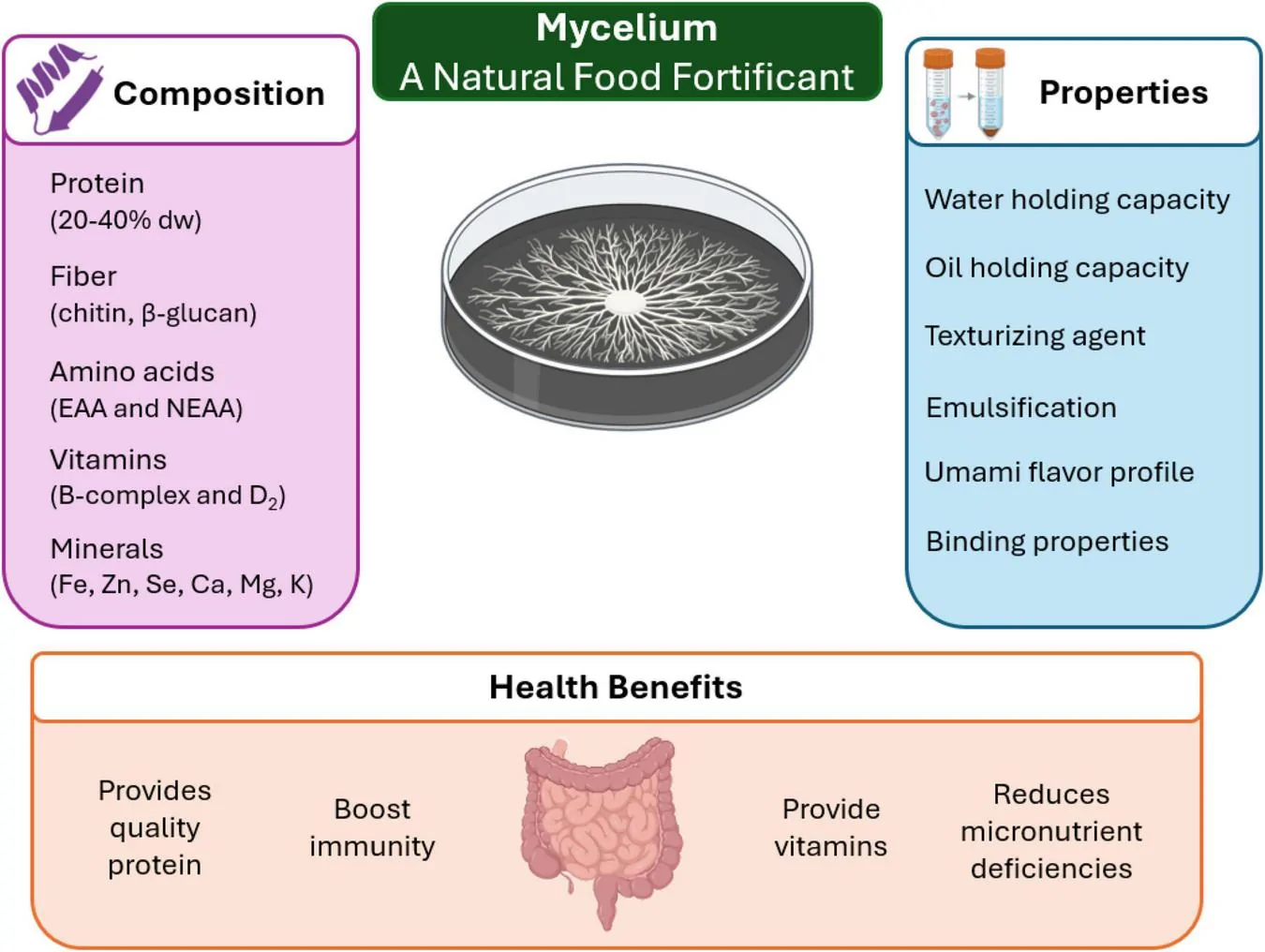
Mycelium as a natural food fortificant: composition, properties, and health benefits.

Mycelium protein differs from traditional plant and animal protein sources due to its unique and complex nutritional composition. Mycelium, the vegetative body of filamentous fungi, is characterized by high-quality complete protein, structural dietary fiber in the form of beta-glucans and chitin, low total fat with a favorable polyunsaturated fatty acid composition, and a mineral-dense ash fraction reflecting active accumulation of iron, zinc, selenium, phosphorus, and potassium from the growth substrate (9, 10). Table 1 presents the nutritional composition of mycelium and compares it with other plant- and animal-based proteins. Mycelium biosynthesizes a range of bioactive compounds, such as ergosterol, ergothioneine, triterpenoids, and immunomodulatory polysaccharides, in addition to macronutrients. These compounds collectively offer functional health advantages that go much beyond basic sustenance (37). Importantly, mycelium is a highly versatile nutritional platform for applications such as functional foods and food fortification, as its nutritional profile can be significantly altered by species selection, substrate modification, and fermentation mode. Mycelium as a food fortificant, its composition, properties, and health benefits are shown in Figure 3.

**TABLE 1:** Nutritional profile of mycelium and its comparison with animal-based and plant-based proteins.

Nutrient	Mycelium *(P. eryngii)*	Wheat gluten	Pea protein isolates	Soy protein isolates	Beef	Egg	Chicken breasts (boneless skinless)	Chicken patties (quorn)
Water (g)	5.73	–	–	–	54.6	75.8	74.8	–
Protein (g)	25.45	66.7	77.8	83.3	11.7	12.4	22.5	12
Lipid (g)	1.72	0	5.56	2.08	28	9.96	1.93	9.33
Fiber (g)	13.01	0	3.7	0	0	0.75	0	2.7
Carbohydrates (g)	47.82	22.2	7.41	0	2.89	0.96	0	26.7
Iron (mg)	41	4	23.3	12.5	1.14	1.67	0.35	0.48
Sodium (mg)	136	0	963	708	872	129	66	467
Calcium (mg)	255	0	74	804	15	48	4	0
Selenium (ug)	331	0	0	0	0	31.1	0	0
Potassium (mg)	–	56	–	58	343	132	330	0
Cholesterol (mg)	0	0	0	0	78	411	73	7
Energy (kcal)	360	333	370	375	310	143	106	227
Reference	Mandliya (113)	USDA 2102078	USDA 2477360	USDA 2417894	USDA 323121	USDA 748967	USDA 2646170	USDA 2138348

### 2.1 Macro and micronutrient

Fungal mycelium has become a nutrient-dense biomass that is characterized by low lipid content, structural dietary fiber, high-quality protein, and an exceptionally rich mineral matrix. Species, substrate, and fermentation technique significantly affect the compositional heterogeneity of fungal mycelium. According to recent research, the protein concentration of food-grade mycelial biomass ranges from 25% to 46% dry weight, placing mycelium in a similar position to legume protein concentrates and, in certain strains, approaching animal protein sources. *Hericium erinaceus* mycelium reached a lysine-rich profile with approximately 42.5% protein, which was beneficial for grain enrichment, while submerged *Pleurotus ostreatus* and *Lentinula edodes* mycelia were found to contain 28.4%–35.7% protein, which makes up more than 40% of the total amino acids as essential amino acids (9, 10, 38). These values reflect the composition of the mycelium rather than its actual bioavailability or protein utilization, especially because chitin and β-glucans limit enzymatic access to intracellular proteins, reducing true digestibility compared to plant or animal proteins (9). *In vitro* digestibility of mycelium varies from 80% to 90%, such as 80% for *F. venenatum*, 85% for *A. oryzae*, and even 90% for *N. intermedia* after simulated digestion (39). The protein digestibility corrected amino acid score (PDCAAS) value of *F. venenatum* was 0.91, based on an estimated digestibility of 78% (40), with net protein utilization (NPU) and biological value (BV) values of 65% and 84%, respectively, compared to 80% and 85% for milk protein (41). In a comparative study of 15 culinary-medicinal basidiomycetes, mycelial carbohydrates ranged from 42.9% to 83.6%, mainly represented by β-glucan, chitin-glucan complexes, and storage polysaccharides, while lipids were lower (1.8%–6.2%), mainly rich in linoleic and oleic acids (42). Importantly, Holt et al. (9) identified whole-food mycelium as naturally containing fiber-protein matrices associated with improved digestibility and reduced antinutritional burden compared to several plant proteins.

Beyond its proximate composition, mycelium plays an increasingly recognized role as a mineral-dense ingredient, particularly for the accumulation of potassium, phosphorus, magnesium, iron, zinc, selenium, and copper. Original quantitative studies have reported potassium concentrations in mycelia from 16.7 to 38.6 g/kg, phosphorus from 1.1 to 11.7 g/kg, calcium up to 7.3 g/kg, and zinc levels reaching 97 mg/kg depending on species and cultivation medium (42). Similar to protein composition, these values reflect mineral composition rather than biaccessible content and do not necessarily indicate nutritional value. For example, the *in vitro* bioavailability of iron in mycelial biomass varies from 44% to 94% depending on the species and cultivation media (43). A multi-species analysis showed ash content ranging from 1.27 to 10.08 g/100 g, with several mycelial forms exhibiting higher mineral density than fruiting bodies, particularly in Fe, Mn, and Zn (44). More recent studies introduced the concept of “biofortified mycelium,” in which selenium-enriched *P. ostreatus* and *P. eryngii* mycelia accumulated 8 and 15-fold higher organic Se than non-fortified controls, respectively, while preserving growth and protein yields, representing a novel functional nutrition strategy (21). Similarly, iron-rich submerged mycelia grown in fortified media showed significantly greater Fe bioavailability than in conventional plant matrices. In addition, ergothioneine-rich mycelium provides sulfur-related micronutrient value, adding a dimension beyond traditional mineral supplements (38). Current evidence positions mycelium not only as an alternative protein source but also as a structurally unique whole-food matrix that combines macronutrients with bioavailable minerals. New comparative studies also show that mycelial biomass often has higher ash, zinc, and phosphorus levels but lower fat content than the corresponding fruiting bodies. At the same time, kinetic-driven modulation allows for the engineering of nutrients suitable for food applications.

### 2.2 Amino acid profile

Recent studies have indicated that fungal mycelium has a nutritionally competitive, often highly balanced amino acid composition, with many taxa approaching or exceeding reference patterns for essential amino acids (Table 2). Comparative compositional analysis of edible mycelia by Berger et al. (10) showed that submerged mycelia of *Pleurotus* and *Lentinula* contained protein fractions rich in glutamic acid and aspartic acid, with biological values ranging from 66 for *L. edodes* to 82 for *Pleurotus* spp. As per FAO/WHO reference, widely used plant proteins such as bean (58) and soy (74). Recently, Zwinkels et al. (36) showed that commercially cultivated basidiomycete mycelia exhibited high-quality protein signatures with PDCAAS ranging from 0.93 to 1.20 and protein content of 33.2 g/100 g dry weight, suggesting them as an “Excellent source of protein” as per the FAO standards, with even four species having PDCAAS over 1.0, indicating complete essential amino acids present in them. Furthermore, mycelial amino acid composition is not constant but strongly modulated by the cultivation substrate and kinetic mode, providing a new avenue for tailoring amino acid enrichment through biological process engineering. A major recent advance is the recognition that substrate-directed cultivation can significantly enhance mycelial enrichment in essential amino acids for food applications. In solid-state kinetic systems, Jia et al. (45) showed that *Pleurotus pulmonarius* mycelium cultivated on wheat bran achieved a crude protein content of 31.2%, a 93% increase over the unfermented one, and an essential amino acid index (EAAI) of 100.20, indicating an exceptionally balanced essential amino acid distribution for food/feed use.

**TABLE 2:** Essential amino acid (EAA) profile of different protein sources (g amino acids per 100 g) (adapted from (114), licensed under CC BY 4.0 and USDA).

EAA	Animal-based protein		Plant-based protein		Mycoprotein	Egg whole
	Beef	Cow milk	Soy isolate*	Soy concentrate*		
Histidine	0.30	0.09	0.6	0.4	0.39	0.28
Isoleucine	0.87	0.20	1.1	0.8	0.57	0.62
Leucine	2.53	0.32	1.8	1.3	0.95	1.05
Lysine	1.60	0.26	1.4	1	0.91	0.83
Methionine	0.50	0.08	0.3	0.2	0.23	0.42
Phenylalanine	0.76	0.16	1.1	0.9	0.54	0.66
Tryptophan	0.22	0.05	0.3	0.2	0.18	0.17
Threonine	0.84	0.15	0.8	0.7	0.61	0.59
Valine	0.94	0.22	1.1	0.8	0.60	0.73
Reference	Derbyshire (114)					USDA 748967

*Soy isolate and concentrate data have been adjusted to the same water content as mycoprotein.

**TABLE 3:** Bioaccumulation/Biofortification of micronutrients during the growth of mycelium.

Micronutrient	Fungal species	Method used	Process parameters	Media	Final micronutrient content	References
Selenium	*Pleurotus ostreatus*	SmF	Temperature 26 °C pH 5.97 Days 7 Agitation 120 rpm; inoculum size 6% (v/v) Growth stimulator 2,4-D (2,4-dichlorophenoxyacetic acid)	Potato dextrose (26 g/L) with yeast (4 g/L) supplemented with Na_2_SeO_3_ at 30.65 mg/L and 2,4-D at 2 mg/L	Organic Se content of 594.85 mg/kg dw in mycelial biomass, i.e., 8.03-fold higher than control; other minerals also increased significantly (Fe from 52.56 to 75.23, Cu from 5.33 to 12.83, Zn from 66.71 to 87.01, and Mn from 4.37 to 6.42 mg/kg dw)	Guan et al. (21)
Selenium and Zinc	*P. florida, P. sajorcaju, P. djamor*	SmF and SSF	SmF: PDB; 25 °C for 20 days inoculated with mycelium of 8 mm size SSF: wheat straw as substrate; 25 °C, pH 5.5–6.5 with relative humidity of 80%	SmF: PDB enriched with Se, Zn and Se-Zn at different concentrations (0, 4, 6, 8, 10, 12 mg/L). SSF: wheat straw (non-seleniferous) supplemented with optimized Se and Zn salt concentrations. Se-enriched wheat straw from Punjab was also tried	Fresh mycelium biomass content increased from 10% approximately with Se and Zn individually, as well as when used in combination, with the control having 60.47 g/L (*P. florida*), 85.97 g/L (*P. sajorcaju*), and 80.66 g/L (*P. djamor*); Individual Se improved yield much higher than Zn alone or when used in combination with Zn	Madaan et al. (15)
Selenium, zinc, and iron	*Flammulina velutipes*	SmF	Standard submerged fermentation conditions for *F. velutipes* with shaking flask	Selenium, zinc, and iron from 0 to 120 mg/L in PDB medium with individual and combination tested	The highest Se accumulation occurred when Se (60 mg/L) and Zn (120 mg/L) were applied together. This resulted in an 85-fold increase in Se content compared to mycelia treated with Se alone, and an 88-fold increase compared to untreated mycelia.	Ramezannejad et al. (85)
Selenium and zinc (zinc sulfate vs. zinc hydroaspartate)	*Pleurotus eryngii*	SmF for mycelium and SSF for fruiting body	SmF: Temperature 25 °C, 16 h light, 8 h dark photoperiod, 28 days cultivation in 500 mL flask with 250 mL volume, and an inoculum size of 0.1 g	Na_2_SeO_3_ (50 mg/L). ZnSO_4_ (87.2 mg/L). Zinc hydroaspartate (100 mg/L)	Zn content increased from 15.1 to 289.5 mg/100 g dw (19-fold increase) with zinc sulfate, 178.3 mg/100 g dw (12-fold increase) with zinc hydroaspartate; Se content increased from 2.1 to 18.79 mg/100 g dw (9-fold increase)	Zięba et al. (87)
Iron	Seven fungal species (*L. crinitus*, *G. lucidum, S. commune*, *P. ostreatus*, *P. eryngii*, *L. edodes*, *and A. subrufescens*)	SmF (two medias – malt extract and sugarcane molasses	Temperature 28 °C, 21 days duration without agitation under no light with 250 mL filled up to 150 mL	Malt extract and sugarcane molasses-based medium (20 g/L) with iron content of 0.116 and 91.23 mg/L, respectively (iron is naturally present in both and no additional supplementation was done)	Iron content in malt extract grown samples ranged from 106 to 213 mg/kg dw based on species; iron content in sugarcane molasses grown samples ranged from 358 to 1304 mg/kg dw across all species. Highest Fe iron was found in *P. ostreatus* (1,304 mg/kg dw). Highest biomass growth in *P. eryngii* (2.31-fold than malt extract)	Scheid et al. (43)
Iron and zinc (individually)	*P. ostreatus* (4 strains), *L. edodes* (2 strains), *P. eryngii*, and *S. commune*	SSF	Temperature 28 °C for 28 days under dark conditions in 90 mm petri dishes inoculated with 5 mm diameter malt extract agar (MEA) containing mycelia	MEA (20 g/L) supplemented with FeSO_4_ at 50 mg/L; and ZnSO_4_ at 7.5 mg/L; control without any iron or zinc addition	Highest iron bioaccumulation was found in *P. ostreatus* (U2-9) = 3197.7 mg/kg dw (control 8.90 mg/kg dw); highest zinc bioaccumulation was found in *P. ostreatus* (U6-8) = 440.4 mg/kg dw (Control 37.06 mg/kg dw)	Umeo et al. (86)
Zinc	*Flammulina filiformis* (2 strains: cultivated FV8 and wild FV18)	SSF	Temperature 25 °C for 14 days	PDB supplemented with ZnSO_4_.7H_2_O at four concentrations (0, 10, 20, and 30 mg/L)	Control (FV8 = 3.6 mg/kg; FV18 = 3.7 mg/kg) At 30 mg/L Zn: FV8 = 50.4 mg/kg; FV18 = 66.6 mg/kg An increase in 14–18-fold was observed depending on strain	Qu et al. (65)
Vitamin D_2_	*Pleurotus sapidus* (strain 8266)	SmF + post-harvest UV-B treatment	Temperature 24 °C for 4 days at 150 rpm in 200 mL malt extract medium in a 500 mL flask followed by UV-B irradiation from 0 to 60 min at 25 °C	Standard PDB medium; no mineral supplementation; photochemical conversion to vitamin D_2_ using UV-B irradiation	Optimal UV-B conditions (10 min exposure at 25 °C) and optimized vitamin D_2_ content increased to 365 μg/g dw from initial value of 3.75 mg/g dw	Ahlborn et al. (11)
Minerals (Fe, Co, and Ca), and Vitamin D_2_	*Pleurotus eryngii*	SmF + UV-B irradiation	Temperature 28 °C for 10 days incubation time at 120 rpm agitation speed with an initial pH of 6 and inoculum volume of 4%, followed by UV-B irradiation post-harvest	A combination of soya flour extract media (20 g/L soya flour extract, 20 g/L sucrose, 3.0 g/L yeast extract) and Jaggery-peptone (VSPe) media (20 g/L jaggery, 4 g/L peptone, 1.0 g/L KH_2_PO_4_, 0.8 g/L KH_2_PO_4_, 0.3 g/L MgSO_4_.7H_2_O, 0.4 g/L CaCl_2_.2H_2_O), and 1.0 ml/L of trace element (TE) solution (FeSO_4_.7H_2_O, 0.5 g/L; ZnSO_4_.7H_2_O, 1.4 g/L; MnSO_4_.H_2_O, 1.6 g/L; COCl_2_. 2H_2_O, 2 g/L)	The mineral composition of Fe, Zn, Co, and Cu were found to be 79.34, 32.64, 10.09, and 1.12 μg/g dw, respectively. Vitamin D_2_ content was found to increase from 4.53 to 320 μg/g dw	Singh et al. (81)

SmF, submerged fermentation; SSF, solid-state fermentation; PDB, potato dextrose broth; PDA, potato dextrose agar; dw, dry weight basis; C:N, carbon to nitrogen ratio; UV-B, ultraviolet-B radiation (290–315 nm). All micronutrient content values are expressed on a dry weight basis for mycelial biomass.

### 2.3 Bioactive compounds

Fungal mycelium has emerged as a metabolically active reservoir of structurally diverse bioactive compounds, often accumulating higher concentrations of some metabolites than the fruiting body under controlled cultivation. Recent studies have revealed β-glucans, heteropolysaccharides, ergothioneine, and fungal immunomodulatory proteins (FIPs) as potent mycelial bioactives with high nutraceutical relevance (34, 46). In submerged *Ganoderma lucidum* mycelia, optimized cultivation resulted in a ganoderic acid concentration of 23.04 mg/g dry cell weight and a total yield of 496 mg/L, demonstrating the potential of the mycelium as a scalable source of triterpenoids (47). Similarly, a recent analysis of medicinal mushroom mycelia grown on by-products as substrates reported β-glucan concentrations ranging from 7.35 to 34.3 g/100 g dry matter and ergosterol from 21 to 104 mg/100 g dry matter, highlighting the importance of substrates and species both in optimizing the production process (48). Ergothioneine-rich mycelia have attracted considerable attention due to their extraordinary redox stability and cytoprotective activity. Mycelial biomass is an excellent food-grade source of this sulfur-containing antioxidant compared to many traditional plant matrices (49, 50). In addition to primary polysaccharides, mycelium biosynthesizes compounds that give rise to low-molecular-weight secondary metabolites, which are increasingly recognized for their functional importance (51). Mycelial triterpenoids (ganoderic acid, lucidanic acid), diterpenes, phenolic acids, and nucleoside derivatives such as cordycepin showed significant compositional modulation in fermentation parameters, the carbon/nitrogen ratio, and the elicitor-supplementation response (52, 53). In *Cordyceps militaris* mycelium, cordycepin enrichment has been correlated with metabolic pathway engineering, while *Ganoderma* mycelia have demonstrated increased accumulation of terpenoid fractions with antioxidant and anti-inflammatory activities under oxygen-limited fermentation (54, 55). Importantly, recent studies have shifted the focus from mere quantification to structure-bioactivity relationships, demonstrating that the branching degree and molecular weight distribution of mycelial β-glucans directly influence immunomodulatory potential, while protein-bound polysaccharides from *Trametes* and *Lentinula* mycelia have demonstrated bioactivity not replicated by pure glucans alone (56, 57).

The recognition of mycelium as a customizable biofactory for food-functional ingredients, where substrate-driven biotransformation can enrich targeted compounds such as phenolics, antioxidants, and neuroactive metabolites, has been widely recognized (33). Recent studies on food-grade mycelial fungi have shown that agro-industrial substrates can significantly alter the phenolic fingerprint, increase antioxidant capacity, and promote the co-production of bioactive and proteinaceous components, opening opportunities for versatile food applications (46, 58). Emerging evidence also points to synergistic interactions between mycelial metabolites. For example, the co-occurrence of β-glucans with ergothioneine and glutathione in cultured mycelia has been proposed to produce multi-targeted antioxidant and immunoprotective effects that are greater than those of the individual compounds alone (49, 50). This systems-level approach offers innovation beyond traditional flavor chemistry, positioning the mycelium not only as a source of individual bioactives but also as an engineered matrix of interacting functional compounds with direct implications for the development of next-generation functional foods, precision medicine, and nutraceutical ingredients.

## 3 Production techniques for mycelial biomass

**FIGURE 4:**
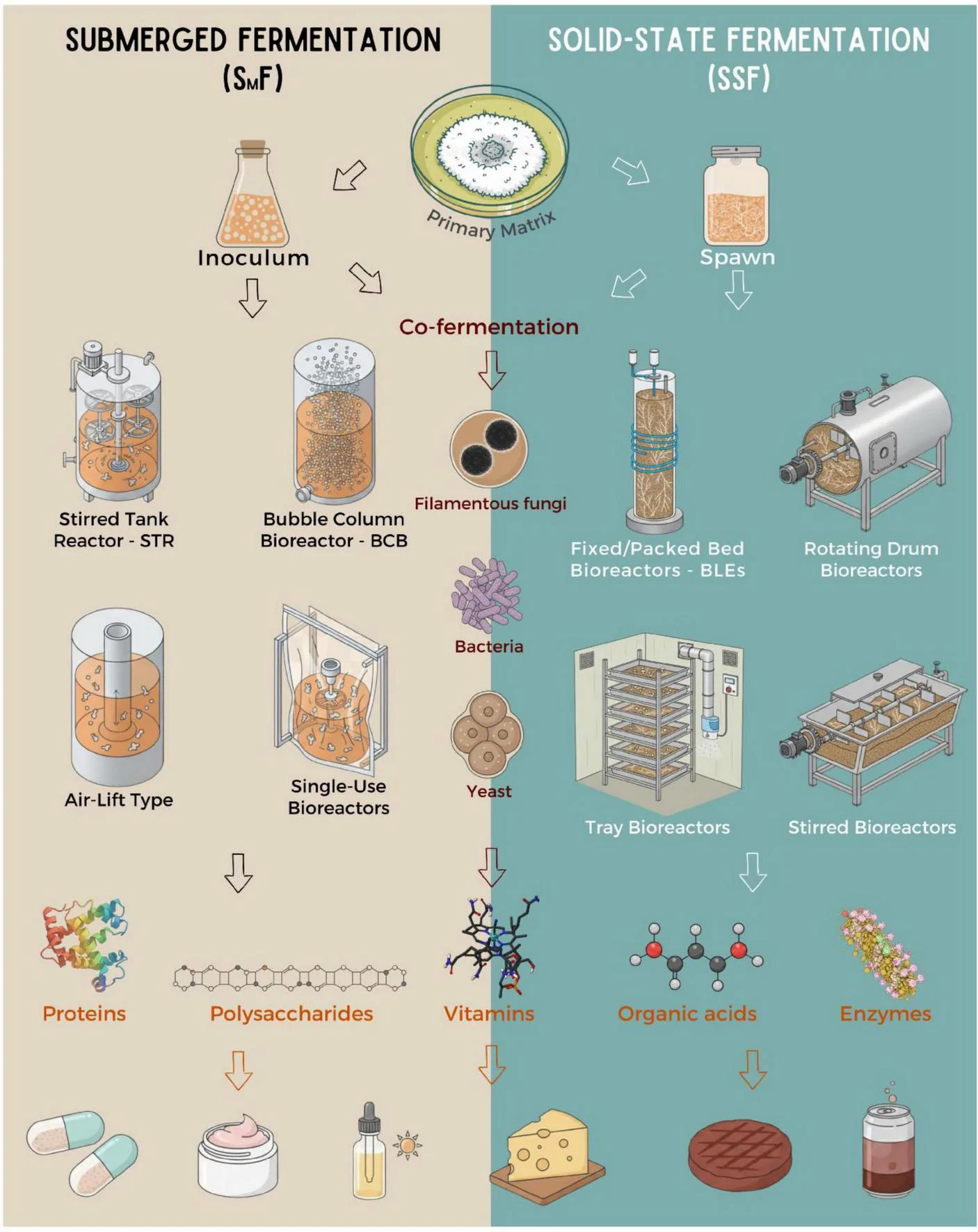
Submerged and solid-state fermentation for mycelium production and its application in food product development. Reproduced from (32), licensed under CC BY 4.0.

The production of high-quality mycelial biomass enriched with micronutrients and bioactive compounds requires a suitable fermentation method and controlled fermentation conditions. Submerged, solid-state, and precision fermentation are the most widely used techniques for the production of mycelium. Each fermentation technique has its unique advantages and disadvantages regarding flexibility of conditions, product quality, moisture content, yield, cost, substrate suitability, and scalability, and thus selecting the appropriate method is important depending on the final output required. A comparison between submerged and solid-state fermentation techniques with their applications is shown in Figure 4.

### 3.1 Submerged fermentation

In SmF, filamentous fungi are cultivated in a liquid nutrient medium, requiring precise control over fermentation parameters to ensure cultural homogeneity and optimal yield (59). Key process parameters such as pH, temperature, aeration, and agitation influence cell morphology, metabolic activity, and nutrient uptake. Most fungal species grow optimally at mildly acidic pH (4–5.5) and at temperatures between 20 °C and 30 °C; thus, controlling pH and temperature within these ranges might be helpful for biomass accumulation or exopolysaccharide (EPS) production. Deviations from this pH range may cause a significant drop in production yield and alter protein and chitin composition (60). Additionally, aeration and agitation are equally important, as they influence oxygen availability and mass transfer, which are essential for fungal metabolism, and may also govern broth rheology. Cui et al. (61) observed that yields of biomass and EPS declined with increasing aeration rate and decreasing agitation rate. In addition to biomass yield, these parameters also affect the accumulation and bioavailability of micronutrients in the mycelium. For instance, Guan et al. (21) optimized the SmF conditions (pH 5.97 and 30.65 mg/L selenium) to receive the highest selenium accumulation (594.85 mg/kg) and biomass yield (15.75 g/L) compared to the control sample. This demonstrates that adjusting the same process parameters that influence biomass can also enhance micronutrient enrichment (see section “4.2 Biofortification approaches”).

Furthermore, the nutritional environment in the bioreactor, for instance, nitrogen and carbon sources, also influences the production of mycelium. For instance, adding 5% glucose and xylose to the fermentation medium yielded different results for EPS and biomass; glucose increased EPS yield, whereas xylose increased biomass production in *Antrodia cinnamomea*. In the case of nitrogen sources, organic nitrogen sources are reported to be a superior choice for mycelium biomass production than the inorganic nitrogen sources (62). Inoculum volume is another factor that affects biomass and metabolite formation. Several studies demonstrated that optimization of inoculum percentage is required to achieve the highest yield. Xu et al. (63) reported that 2% inoculum volume increased the yield of biomass and EPS of *Armillaria luteovirens*, and adding more or less inoculum decreased the yield significantly. In another study, inoculum volume varied between 0.5% and 6.0 % to ascertain the effect of mycelial biomass production by edible mushroom *Pleurotus albidu* and found that increasing the inoculum percentage increased the yield (64). Hence, it can be inferred that the inoculum volume required to obtain the highest biomass yield depends on the fungus culture in SmF. The inoculum size and nutrient supplementation jointly influence mineral uptake efficiency. For instance, Qu et al. (65) studied the effect of zinc supplementation on biomass yield and found that biomass yield in *Flammulina filiformis* increased by 15%–18% for 20 mg/L zinc addition, and zinc accumulation increased by 14–18 times with 30 mg/L zinc addition. This study shows that biomass yield and micronutrient accumulation are interrelated rather than independent variables.

### 3.2 Solid-state fermentation

Solid-state fermentation is a waste-recycling process in which microorganisms, such as filamentous fungi, yeasts, and bacteria, cultivate on insoluble solid substrates in the absence of free water or with low water content (66). In this process, microorganisms are provided with lignocellulosic plant material on a solid, moist surface to simulate natural growth conditions, thereby increasing fermentation efficiency, improving product stability, and reducing catabolic suppression and bacterial contamination. Furthermore, SSF can produce bioactive compounds with higher enzymatic activity in the absence of organic solvents, at lower operational and capital costs, making it suitable for industrial applications. Further, in comparison to SmF, SSF requires lower energy inputs, generates less wastewater, demands less sophisticated infrastructure, and allows the valorization of low-cost agro-industrial by-products, including rice straw, wheat bran, sugarcane bagasse, coffee husks, brewery spent grains, and soybean hulls, as fermentation substrates (67). In addition to their role in substrate valorization, SSF conditions are directly linked to micronutrient outcomes. For example, wheat straw substrates enriched with selenium and zinc, cultivated under optimized SSF conditions (pH 5.5–6.5, 80% RH), exhibited higher fresh mycelial biomass and mineral enrichment levels similar to those in submerged systems (15). These findings emphasize that SSF process parameters can be adjusted for micronutrient enrichment, beyond merely producing biomass.

Solid-state fermentation has distinct potential to improve the nutritional value, digestibility, and overall quality of plant-based materials. For instance, some authors reported that SSF of plant substrates, including wheat bran, quinoa, oat, etc., with edible fungi, such as *P. ostreatus, Pleurotus eryngii*, and *P. pulmonarius*, showed improved protein content, water holding capacity, gelling properties, and oil absorption capacity and diminished anti-nutritional factors such as phytic acid and tannins (45, 68). Likewise, SSF of whole wheat and rice with medicinal *Ganoderma* species showcased increased protein, vitamins, dietary fiber, and bioactive compounds (69, 70). It is important to note that SSF has also been reported to be a better choice for improving the sensory and textural properties of *Rhizopus oligosporus* biomass than SmF (71). However, SSF-derived mycelium from species such as *Monascus purpureus*, a filamentous fungus responsible for the traditional production of crimson yeast rice in East Asia, showed around 14% reduction in protein content compared to SmF (72).

Considering that SSF offers an active and advantageous alternative for mycelium production, the major challenges remain in finding suitable microorganisms and their substrates, optimizing process parameters, and scaling up laboratory-based techniques. Further challenges in the purification of products in the downstream process and in minimizing variability between batches remain unaddressed (73). Therefore, to address these constraints, biochemical researchers have been actively working on developing particular types of bioreactors (rotating disk, rotating drum, fixed bed, column, tray, fluidized bed, air pressure pulsation, etc.) that can improve the overall quality of the mycelium intended for whole-food or co-ingredient applications (74).

### 3.3 Precision fermentation

Precision fermentation is an advanced fermentation method in which bacteria, fungi, and yeast are genetically modified to produce desired products, such as rennet and citric acid. More precisely, this is an interdisciplinary approach that integrates synthetic biology and various fermentation techniques with genetic engineering to produce mycoprotein with improved nutritional profiles, greater production efficiency, and reduced environmental footprints (75). This process has made a substantial footprint in the food industry as an alternative means of producing next-generation protein ingredients without using natural sources, such as animals and plants (76), but producing proteins similar to casein or albumin in taste and texture. It is projected that, soon, the production of alternative proteins via precision fermentation will be much more efficient in terms of land use, feedstock utilization, time management, and water use (77). Presently, this technique is primarily used in the production of milk and egg proteins, but is gaining attention for the production of specific meat analog components.

A study conducted by Tong et al. (20) applied metabolic engineering in *F. venenatum* for the production of mycoprotein and found that the process could enhance the protein content in mycoprotein by 57%, improve the carbon conversion ratio by 62%, and mycoprotein synthesis by 57%, as well as significantly reduce CO_2_ emission. In connection with this work, Wu et al. (19) used the CRISPR/Cas9-mediated scarless gene knockout technique to develop *F. venenatum* with improved nutritive value and a higher glucose conversion rate, targeting two key genes, namely chitin synthase and pyruvate decarboxylase, and compared with alternative proteins. It was observed that the engineered *F. venenatum* strain enhanced the essential amino acid index and mycoprotein production rate by 32.9% and 88.4%, respectively. Earlier, precision fermentation was mostly used to improve protein yield and carbon efficiency. However, with the application of metabolic engineering techniques, precision fermentation has evolved to produce specific bioingredients, including micronutrients, directly rather than producing them in substrates (78). Moreover, life cycle analysis of the process showed potential to reduce global warming by 4%–61.3%, depending on the national production scenario. In a parallel study, the CRISPR/Cas9-mediated homology-directed recombination system was employed in *F. venenatum* to simultaneously produce edible mycoprotein and the red food colorant betanin. The results showed that fermentation yielded 1.91 and 9.53 g/L of betanin and mycoprotein, respectively, within 48 h. Simultaneously, the pH naturally decreased from 6 to 4 without any acid addition, demonstrating the potential of precision fermentation technology to produce multifunctional mycelial biomass with enhanced nutritional, sensory, and commercial value (79). Although substantial work is in process for the production of alternative protein using the precision fermentation technique, integration of artificial intelligence-based predictive modeling for process optimization and the integration of IoT sensors in bioreactor engineering for monitoring and precisely regulating pH, temperature, dissolved oxygen, and biomass concentration are required for process consistency and successful scale-up processes.

It is crucial to emphasize that, when combined, these three methods for producing mycelial biomass can build a strong, scalable, and advanced biotechnological system that can sustainably meet the world’s demand for premium mycelial biomass.

## 4 Strategies for micronutrient enrichment

**FIGURE 5:**
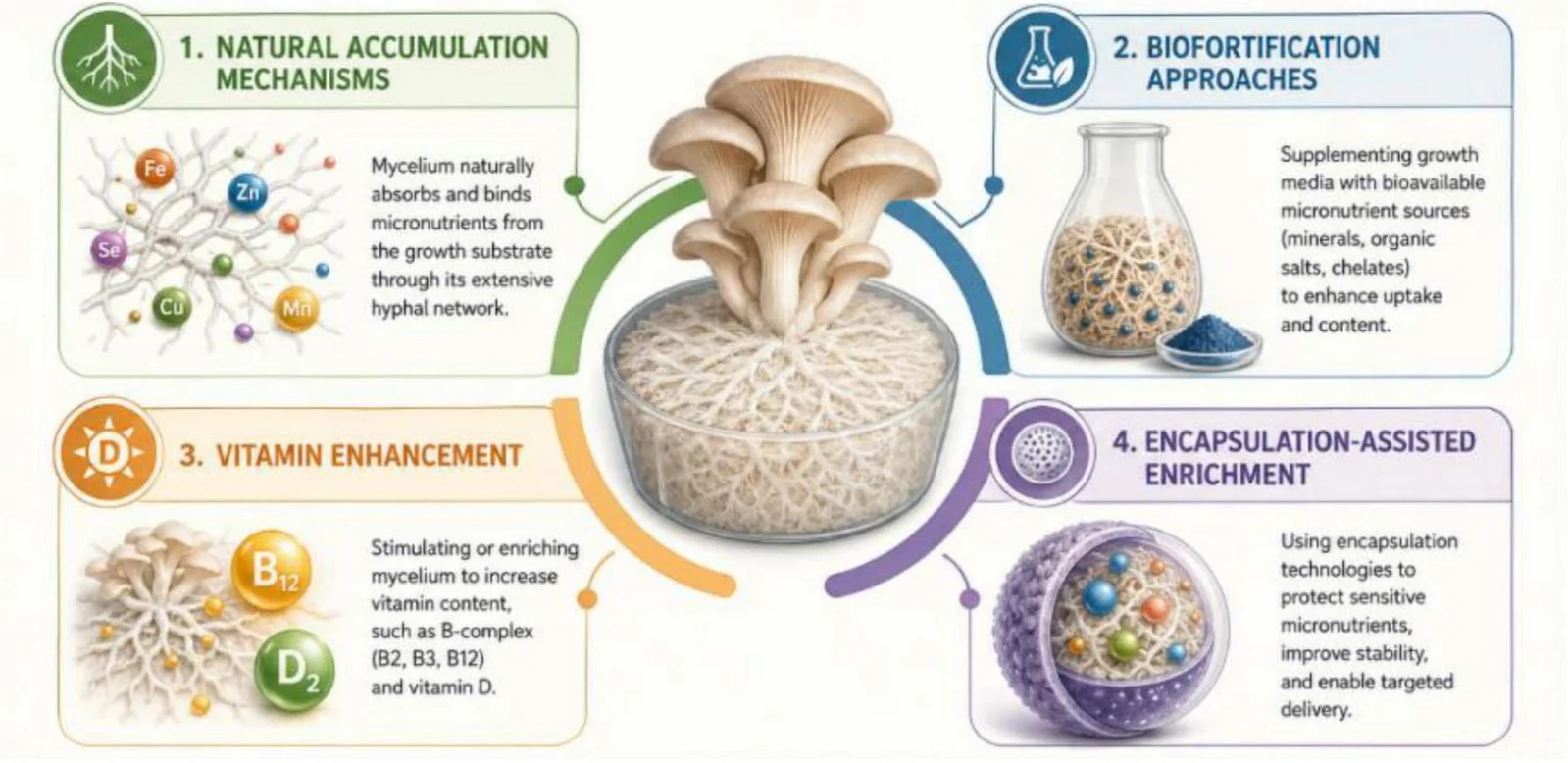
Schematic diagram showing the strategies for micronutrient enrichment in mycelium. This figure was generated using Open AI Image Generation and the input prompt can be found in Supplementary File 1.

Micronutrient enrichment strategies for mycelium include several complementary approaches to improve the nutrient density and bioavailability of fungal biomass. Natural accumulation mechanisms utilize the inherent capacity of mycelia to take up and biotransform minerals, such as iron, zinc, selenium, and calcium, from growth substrates into bioavailable organic forms (80). Biofortification methods further enhance this process by supplementing substrates, controlling metabolism, and implementing cultivation strategies (8, 15). In contrast, vitamin enrichment strategies focus on stimulating the biosynthesis or exogenous incorporation of vitamins such as the B-complex and ergocalciferol (vitamin D_2_) (11, 81). In addition, encapsulation-assisted enrichment has emerged as an advanced strategy to protect sensitive micronutrients, improve preservation during processing, and enable targeted delivery within the fortified mycelial matrix (82, 83). The various strategies employed for micronutrient enrichment in mycelium are presented in Figure 5. Table 2 shows discusses the bioaccumulation and biofortification of micronutrients (minerals and vitamins) during the growth of mycelium.

### 4.1 Natural accumulation mechanisms

Ion transporters, cell-wall biosorption, intracellular chelation, and conversion into organic micronutrient complexes are recognized as natural mechanisms for micronutrient accumulation in fungal mycelia, constituting a metabolically controlled biofortification process (84). There is an 88-fold increase in selenium accumulation when 60 mg/L selenium and 120 mg/L zinc are added to *Flammulina velutipes* and an 85-fold increase for Se-alone treatment compared to untreated controls, indicating coordinated regulation of metal transport and antioxidant defense pathways (85). Similarly, a low dose (4–8 mg/L) supplementation of selenium enhanced radial growth and biomass of mycelia in three *Pleurotus spp.* (*P. florida*, *P. sajor caju*, and *P. djamor*) at different levels from around 9%–12%. Higher concentrations (10–12 mg/L) gave inhibitory results that are consistent with a hermetic dose-response pattern (15). Evidence suggests that vacuolar compartmentalization and the accumulation of peptides such as metallothionein are central to the long-term intracellular storage of micronutrients, providing a mechanistic innovation beyond the traditional passive biosorption model (80, 84).

A parallel but distinct strategy was used to enrich iron and zinc in the mycelium, which includes metabolic redistribution into enzyme-associated pools, chitin–β-glucan cell wall binding, and cation transport systems. Zinc accumulation increased by 14–18 times at 30 mg/L Zn^2+^ addition in *F. filiformis* mycelia, while lysine content was highest at 20 mg/L Zn^2+^. This process also upregulates lysine biosynthesis genes, indicating a coupling between mineral uptake and amino acid metabolism (65). Similarly, iron accumulation increased to 3197.7 mg/kg when *P. ostreatus* mycelium was grown under 50 mg/L Fe supplementation, which contributed to ferric reductase activity and siderophore-mediated chelation (86). These results show that micronutrient enrichment is genetically regulated, with bioconcentration factors and translocation coefficients as major screening criteria for selecting a superior mycelium platform. These findings showed the journey of simple fortification toward biologically programmed micronutrient accumulation, where substrate mineral speciation, transporter engineering, and synergistic co-enrichment are emerging as natural strategies for producing micronutrient-dense fungal mycelium with improved nutritional functionality.

### 4.2 Biofortification approaches

Fungal mycelium has evolved from simple salt supplementation to integrated metabolic engineering and process-oriented enrichment strategies of micronutrient biofortification that improve the elemental loading and bioavailability. Early submerged fermentation studies demonstrated that mineral-enriched culture media can substantially enhance trace element accumulation in mycelial biomass (8). For example, iron biofortification of basidiomycete mycelia grown on sugarcane molasses resulted in up to 1,304 mg/kg Fe in *P. ostreatus*, a 6.8-fold increase compared to conventional malt extract media. *In vitro* iron bioavailability was 44%–94%, indicating species-dependent bioaccessibility beyond total accumulation alone (43). Recent approaches have been directed toward selenium and zinc co-biofortification, where controlled supplementation under SmF and SSF improved mycelial biomass, mineral uptake, and concomitantly stimulated total soluble proteins, flavonoids, and antioxidant activity in *Pleurotus spp*., highlighting a dual nutritional-functional fortification paradigm (15, 87). Collectively, these studies suggest that optimizing nutrient speciation, dose thresholds, and carbon source composition can modulate fungal transporter activity and intracellular chelation, thereby enhancing the partitioning of micronutrients into organic, bioavailable forms.

Emerging strategies to improve the micronutrient density of mycelium have been increasingly applied, including precision biofortification via response surface optimization, elicitor-assisted fermentation, and multi-mineral fortification (15, 21, 87). The application of Na_2_SeO_3_ and 2,4-dichlorophenoxyacetic acid as growth stimulants in the liquid fermentation of *P. ostreatus* was an important advancement. Under the optimized conditions (30.65 mg/L selenium and pH 5.97), the production of 15.75 g/L biomass and 594.85 mg/kg organic selenium was achieved, which was 8.03-fold higher than that in the controls. In addition, the balance of essential amino acids was improved to better align with FAO/WHO standards (21). Such results suggest a transition from passive fortification to metabolically responsive enrichment, in which micronutrients are not only nutritional targets but also elicitors of biosynthetic pathways. Reports have been focused complementarily on substrate engineering, delivery through nanoparticles, and enhancement of vitamin D_2_ by UV in fungal systems with special emphasis on synergistic enrichment of Fe-Zn-Se and their effect on ergosterol derivatives, polysaccharides, and redox enzymes (8, 80). Such multi-target approaches are increasingly seen as scalable strategies for engineering mycelium as a functional biofactory for micronutrient-dense foods.

A major conceptual innovation in the recent literature is the shift from basic mycelial enrichment to a “nutritional program” that combines biofortification with systems-level control of uptake, intracellular partitioning, and nutrient bioaccessibility. Current strategies are based on the bioconversion of complementary minerals, as these forms are better absorbed and more stable in the gastrointestinal tract than inorganic salts. Sustainable methods such as co-fortification and circular bioprocesses have been developed that use agricultural residues as growth media and inexpensive substrates for micronutrient carriers (88). Looking ahead, targeting fungal chelate biosynthesis and oxidative stress signaling could advance the mycelium biofortification from an experimental approach to a predictive engineering-based platform.

### 4.3 Vitamin enhancement

The primary strategy for vitamin enhancement in mycelial biomass has focused on ergosterol bioconversion to vitamin D_2_ through controlled UV exposure and fermentation engineering. Ahlborn et al. (11) studied submerged cultivation of *Pleurotus sapidus* and showed that endogenous ergosterol was converted to vitamin D_2_ after post-harvest UV-B treatment. Vitamin D_2_ concentrations increased under optimized irradiation regimes from trace amounts to >100 μg/g dry weight, while the functionality of the mycelial protein was retained. This strategy has since evolved beyond simple photoconversion toward precursor-directed metabolic enhancement, in which ergosterol accumulation is first elevated by modulating the carbon: nitrogen ratio, oxygen transfer, and sterol biosynthetic flux, followed by photochemical conversion (11). Recent studies on edible fungal biofortification have further shown that selenium- or zinc-enriched substrates can stimulate antioxidant defense systems in mycelial matrices, highlighting the roles of Se and Zn (8, 87), which, in turn, may help preserve labile and sensitive micronutrients. Such coupling of metabolic precursor amplification with targeted post-processing represents a shift from passive fortification to systems-level vitamin bioengineering in fungal biomass.

One of the most recent strategies for micronutrient enrichment in mycelium is substrate-mediated programming, which induces *de novo* biosynthesis (89). In this regard, supplementing submerged media with vitamin precursors has substantially increased vitamin content, depending on the species and fermentation type (8, 9). Furthermore, vitamins co-supplemented with selenium or iron have better stability during drying and downstream processing. The integration of biofortification with fungal physiology suggests that trace mineral enrichment may not act merely as a nutrient addition but also as a regulator of cofactor-dependent vitamin biosynthetic enzymes. Also, a micronutrient stress signal can regulate folate conversion and the biosynthesis of niacin, producing mycelium with more vitamins and minerals, as seen in *Pleurotus*, *Lentinula*, and filamentous mycelium (8, 87). This dual-enrichment innovation has a significant effect on the development of mycelium-based functional foods, which will address hidden hunger through integrated micronutrient delivery.

### 4.4 Encapsulation-assisted enrichment

Mycelium micronutrient enrichment using encapsulation technology has gained popularity for its biofortification strategy and controlled nutrient delivery. Encapsulation overcomes limitations such as poor uptake efficiency, polyphenols delivery, and instability of micronutrients (82, 83). The substrate-mediated mineral loading, in which iron was used to enrich *basidiomycete* mycelia, showed that micronutrient loading increased when delivered through a protected system. In this regard, Scheid et al. (43) enriched *P. ostreatus* in submerged cultivation with sugarcane molasses, where it accumulated iron up to 1,304 mg/kg. Also, the overall iron yield was increased 2–12-fold, and *in vitro* iron bioavailability reached 94% depending on the species and media composition (43). Based on this, mineral carriers based on encapsulation techniques, such as polysaccharide-coated iron complexes, liposomal Fe/Zn dispersions, and nano-enabled selenium formulations, have been proposed to modulate ion release around growing hyphae, alleviating oxidative stress while improving fungal uptake kinetics. This encapsulation-assisted biofortification concept is particularly novel for mycelium systems because the fungal cell wall (β-glucans/chitin) can combine with encapsulated minerals to form a dual-functional micronutrient reservoir with improved gastrointestinal bioavailability.

Fungal mycelia have not been extensively studied with co-encapsulation techniques for zinc and selenium, but substrate-level co-fortification research offers a solid basis for this method. The selenium fortification at 30.65 mg/L Na_2_SeO_3_ produced mycelia of 15.75 g/L and 594.85 mg/kg organic selenium, representing an 8.03-fold increase compared to the control (21). In the same line, Madaan et al. (15) reported the co-fortification of Zn-Se in Pleurotus spp. showed that flavonoids and proteins increased in a dose-dependent manner, with lower micronutrient concentrations improving biomass productivity and radial growth. Polymers such as chitosan and alginate have been used to encapsulate or co-deliver Zn and Se for controlled release in biological systems, thereby improving stability and release kinetics (90), suggesting a similar approach for mycelium biofortification. These encapsulation methods are functioning as a fortification tool and a metabolic pathway, capable of enriching nutrient and metabolite synthesis. This creates a promising system for engineering micronutrient-enriched mycelium ingredients in which external encapsulation, intracellular fungal biotransformation, and post-harvest mycelial encapsulation operate as a three-tier enrichment model. Such convergence represents a breakthrough for next-generation functional foods, particularly in the design of mycelium-based micronutrient delivery systems that are bioavailable, stable, and multifunctional.

## 5 Application for the development of functional food products

The filamentous fungal biomass represents an emerging alternative source for enrichment of bioactives, such as, vitamins, polyunsaturated fatty acids (e.g., palmitic acid, linoleic acid, and oleic acid), phenolic compounds, and minerals, as well as macronutrients, e.g., dietary fibers (especially, β-glucan) and proteins; thus, contributing for fast digestion in humans and animals (33). The fungal species, for instance, *Aspergillus* and *Fusarium*, contain high levels of essential amino acids, including threonine, lysine, and methionine, which are often limited in plant-based sources like soybeans (91). Animal-originated proteins (especially from meat and dairy sources) are also considered balanced in terms of digestibility and bioavailability. However, their environmental, ethical, and resource-specific issues are driving consumer preference toward fungal-derived proteins in the search for sustainable alternatives (92). Despite the protein status, the availability of bioactive compounds in the composition, such as β-glucan and ergosterol, exhibits enormous physiological health benefits, placing the filamentous mycelium not only as a sustainable protein source, but also as a next-generation ingredient for therapeutic and nutraceutical food formulations (93).

### 5.1 Beverages

The development of functional beverages via the fermentation process offers enhanced nutritional, sensory, and physiological health benefits. Fermented dairy products and beverages continue to expand the functional food markets due to rising consumer demand and the promotion of health benefits. Various mushroom-derived bioactives, including β-glucan, polysaccharides, and mycelium, have been incorporated into the preparation of functional food matrices, such as fermented beverages, kombucha, cheese, and yogurt (32). Ontawong et al. (94) prepared *C. militaris* mycelium using a submerged fermentation process, followed by freeze-drying to produce mycelium powder, and incorporated it into fruit juice. The authors stated that the healthy drink resulted in stimulating the human immune response by activating NK cells, reducing inflammatory cytokine secretion, and increasing monocyte concentrations in men and women without toxicity after 8 weeks of consumption. Wang et al. (95) studied the effect of solid-state fermentation of soybean meal powder in distilled water (in the ratio of 1:1) using three edible mycelia (*F. velutipes*, *H. erinaceus*, and *P. ostreatus*) and observed that the nutritional parameters, such as protein, aspartic acid, glutamate, and total polyphenol content increased significantly by 9.49%, 23.16%, 23.39%, and 235.9%, respectively, which might be due to the generation of enzymes, including cellulases and β-glycosidases with their action of breakdown of macromolecules and liberation of phenolic substances, thus enhancing the antioxidant activity. Escobar et al. (30) formulated functional beverage from whey (12.5 g/L), rice bran (50%), and amaranth flour (50%) using the fermentation process incorporated with mycelium species *Aspergillus oryzae* and probiotic strain *Lactobacillus delbrueckii* and the developed beverage was nutritionally rich (protein: 7.72%) and fiber content: 18.13%) with enhanced carbohydrate availability and microbiologically safe up to 29 days at 4 °C. This phenomenon might be attributed to the production of hydrolytic enzymes by *A. oryzae*, which release the fermentable sugars from cellulose, starch, and hemicellulose present in the rice bran and amaranth flour (96).

### 5.2 Dairy alternatives

The untreated cheese whey, a major by-product of the dairy industry, poses serious environmental risks due to the presence of organic and inorganic loads and elevated biochemical oxygen demand. Additionally, the higher lactose content makes cheese whey and other dairy by-products suitable substrates for microbial fermentation to transform them into high-value dairy alternatives. Kaya et al. (97) evaluated the applications of the fermentation process for producing cheese whey-based protein-enriched fungal biomass, incorporating *Neurospora intermedia*, *Rhizopus oryzae*, and *A. oryzae*, in which the protein content of the substrate was raised by 165.54% with *A. oryzae*. The fungal bioconversion process could effectively produce nutritionally enriched biomass as an alternative food from whey powder.

### 5.3 Bakery products

The mushroom fruiting bodies and mycelia are gaining popularity for their incorporation into bakery food matrices to deliver bioactive compounds (32). However, the mycelia are not consumed directly as food but can be added to formulations as flavoring agents and food ingredients to improve functionality (9). Ulziijargal et al. (31) prepared bread by substituting the 5% wheat flour with mushroom mycelia of *Antrodia camphorata*, *Agaricus blazei*, *H. erinaceus*, and *Phellinus linteus* and observed no adverse effect on the textural properties of the bread and retention of sufficient quantity of ergothioneine (0.79–2.10 mg/g dry matter) in the mycelium incorporated bread after the baking process. Stoffel et al. (29) developed mycoprotein (*Pleurotus albidus*) fortified chocolate cookies by replacing the wheat flour (80 g). The authors found that the use of *P. albidus* mycoprotein flour increased the nutritional value, with enrichment in protein (67%) and dietary fiber (336%), and enhanced functionality (phenolic content: 81% and antioxidant activity: 159% DPPH) compared to only wheat-flour-based cookies. However, replacing 80 g of wheat flour with mycoprotein flour negatively affected the textural and sensory properties of the cookies compared to non-fortified cookies.

### 5.4 Meat analog

**FIGURE 6:**
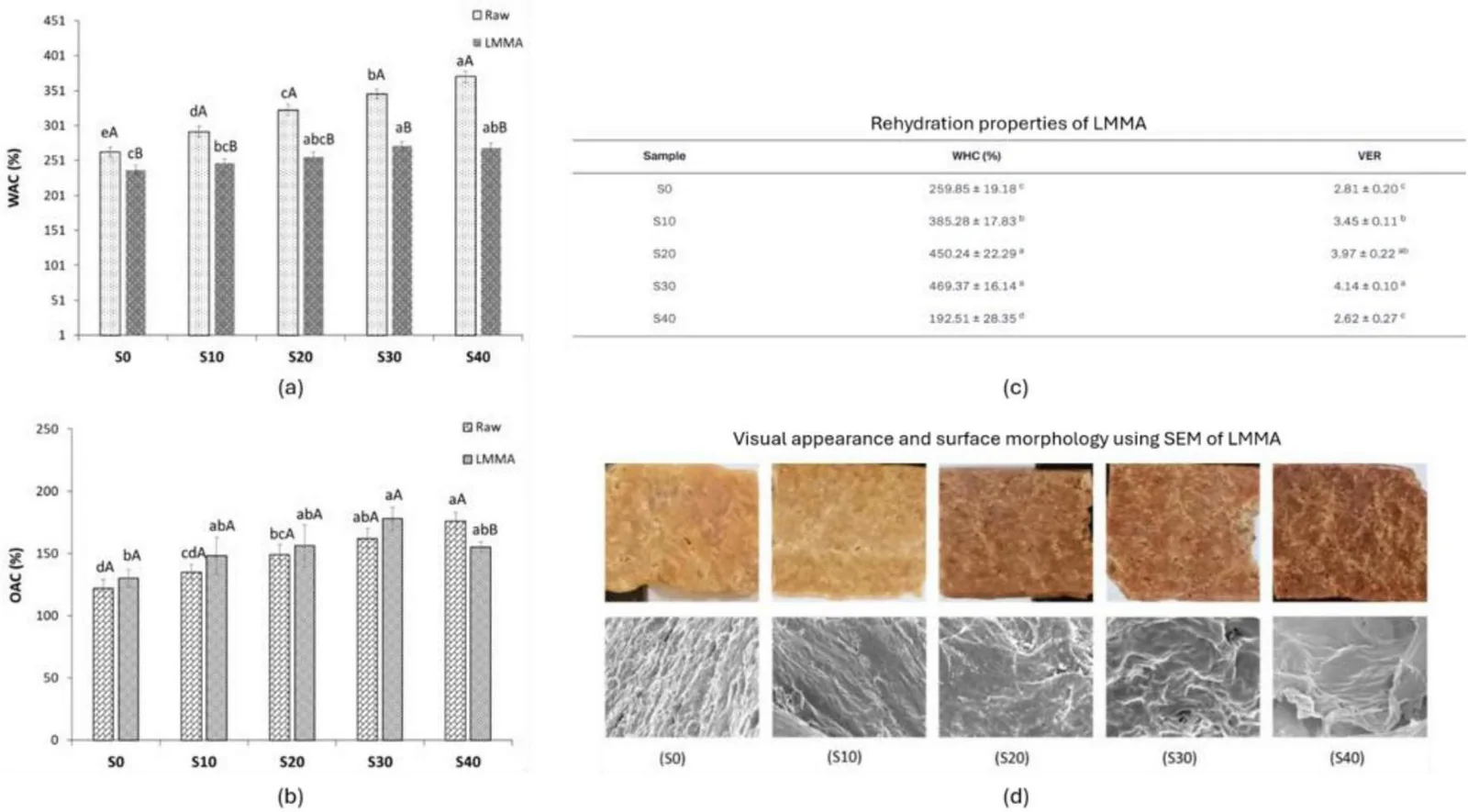
Incorporation of 0%–40% w/w mycelium (*Pleurotus eryngii*) into pea protein isolate to develop low-moisture meat analogues (LMMA). **(a)** water absorption capacity, **(b)** oil absorption capacity, **(c)** rehydration properties [water holding capacity, WHC; and volumetric expansion ratio (VER)], and **(d)** visual appearance (top) and surface morphology (bottom) of the dried LMMA. Reproduced from (101), licensed under CC BY 4.0.

Meat alternatives are structurally modified products that mimic processed meat in terms of texture, appearance, and flavor (98). Mostly, meat analogs are produced by texturization of vegetable proteins, such as pea protein, wheat proteins, and soy protein via spinning, shear, cross-linking, and extrusion processes (99). Mycelium has enormous potential as an alternative source of protein for converting into a meat analog with good mouthfeel and textural consistency (100). Mandliya et al. (101) developed a low-moisture meat analog with the incorporation of mycelium species *P. eryngii* (0%–40% w/w) into pea protein isolate using an extrusion process (at 140 °C die temperature, 40 rpm screw speed, 10 rpm feeder speed, and feed rate of 0.53–0.54 kg/h). The authors observed that the addition of mycelium up to 30% had a minimal effect on the structure, with the enhancement of functional properties resulting in low moisture novel protein-enriched meat analogs (Figure 6). The addition of fibrous and porous mycelium resulted in preservation of the protein network structure, leading to higher water absorption capacity and lower protein solubility of the meat analog (102). Nian et al. (103) studied the effect of the addition of yeast protein on the qualities of the mycelium-based meat analog prepared by the mold filling process. The authors observed that the developed product (up to 10% yeast protein) had protein and fiber contents of 14% and 12.7%, respectively, with superior textural and structural properties. Bartholomai et al. (104) also reported *Neurospora crassa* mycoprotein as a suitable functional mycelium-based ingredient, providing protein, fiber, iron, and potassium with no adverse effect on humans and animals.

## 6 Sustainability and environmental aspects

**FIGURE 7:**
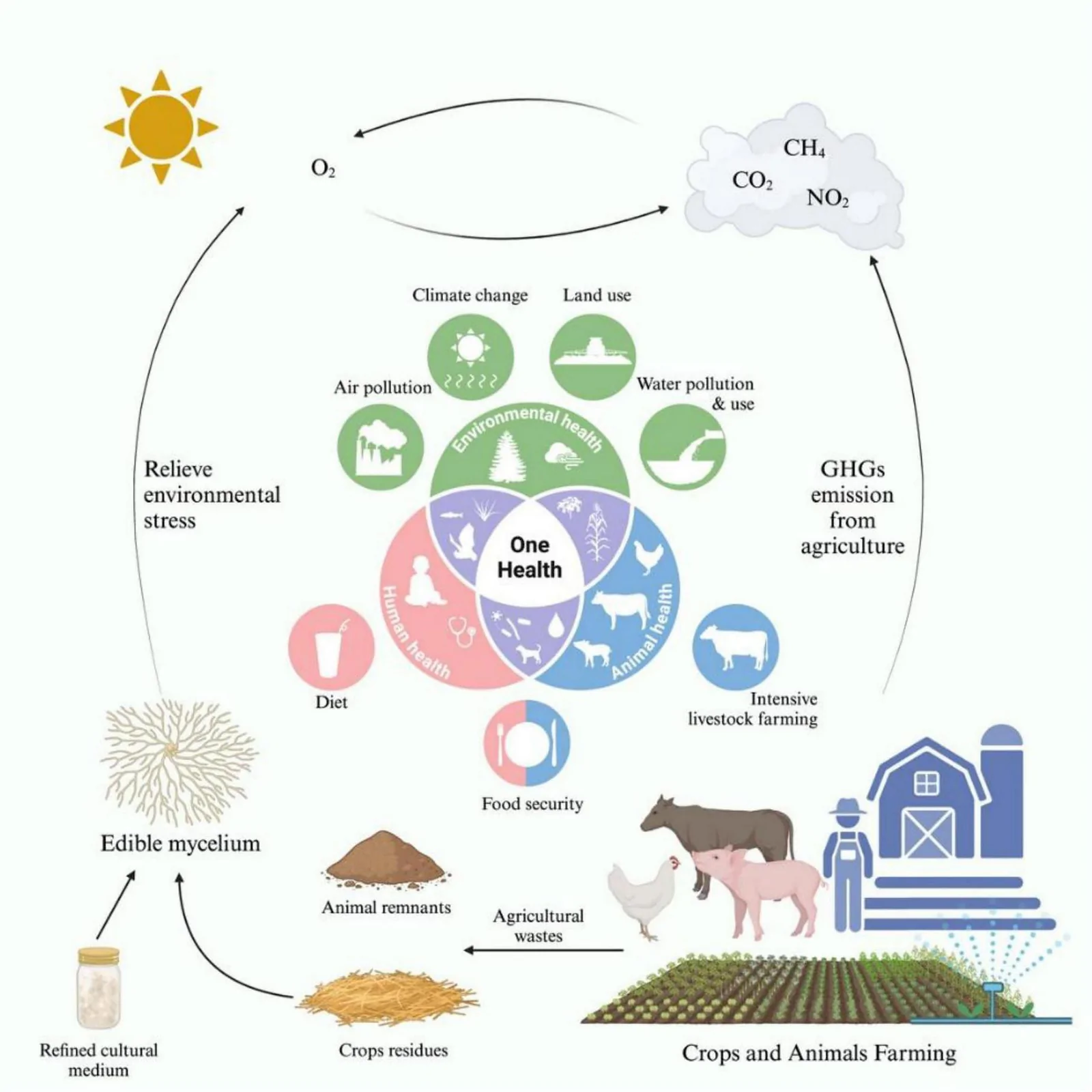
Schematic diagram showing the overview of sustainability and environmental impact of mycelium. Reproduced from (112) with permission from Institute of Food Technologists.

The production of mycelium is much more sustainable and environmentally friendly than animal production, making it a promising alternative for current and future protein sources. A schematic diagram outlining the sustainability and environmental impact of mycelium is presented in Figure 7. According to a recent report by The Carbon Trust (13), mycoprotein production requires 86% less water, 5.5 times less land, and produces around 98% fewer greenhouse gas (GHG) emissions per kilogram of protein than beef, which requires the most resources and generates the highest GHGs of all other animals. Mycoprotein continues to have a distinct environmental advantage over chicken and pork, which are generally regarded as more efficient meat alternatives. Compared to chicken, mycoprotein produces four times fewer GHGs and requires 3.6 times less land per kilogram of protein (13). These studies highlighted the importance of mycelium in developing high-protein products within limited resources and significantly less time than animal protein production.

Conventional fortification in the food industry requires synthetic ingredients or chemicals in concentrated form, such as synthetic iron salts (ferric pyrophosphate, ferrous sulfate, ferrous fumarate, etc.), zinc oxide, or chemically produced vitamins such as vitamin D_2_ or cyanocobalamin. These synthetic fortificants have poor bioavailability and should follow strict guidelines during manufacturing or incorporation into food products, due to their chemical forms, and may pose potential side effects on the human body (105, 106). Their production process involves energy-consuming industrial chemical synthesis and produces waste streams, which contribute to an environmental burden that is often overlooked in fortification policy. Mycelium, on the other hand, has a fundamental advantage by providing nutrient sources, such as vitamins and minerals, such as iron, zinc, selenium, etc., in the media during fermentation, thus bioaccumulating minerals and vitamins from its growth substrate through biological uptake mechanisms, offering the same or superior micronutrient density without synthetic chemical inputs. It can eventually replace synthetic ingredients in the food industry, as it has fewer regulatory guidelines and higher bioavailability due to its naturalness. Mycelium thus positions itself as a clean-label natural fortificant, aligning well with consumers’ demand for ingredient-based, chemical-free, minimally processed, and regulatory-favoring natural-source nutrient enrichment over synthetic supplementation (8, 88).

Even a small shift in dietary protein from beef to mycoprotein has significant global implications. Humpenöder et al. (14) recently modeled four different dietary replacement scenarios between beef and microbial protein. They found that replacing just 20% of per-capita ruminant meat consumption globally with microbial protein by 2050 would reduce annual deforestation rates and related CO_2_ emissions by roughly 56%, while also lowering enteric methane emissions. This effect would be impossible to achieve with static dietary shifts to plant proteins alone due to the non-linear land-use dynamics of ruminant production systems. Another benefit of mycelium is its ability to grow on food industry byproducts and agri-waste streams, including wheat bran, rice straw, sugarcane bagasse, spent brewery grains, and sugar beet pulp, converting low-value organic waste into high-value ingredient rich in minerals and vitamins (17, 18). By converting waste into nutritious value, closing biological cycles, and reducing the environmental effect of micronutrient distribution compared to synthetic fortification techniques, this circular approach connects mycelium biofortification with the concepts of the circular bioeconomy.

Mycoprotein’s environmental impact is further reduced as manufacturing efficiency rises through ongoing technological advancements. By using renewable energy in 2021, Quorn Foods reduced its carbon impact by 30%. They also cut their overall operational emissions by 84% between 2012 and 2023 (13). Depending on the energy source utilized for fermentation, new precision fermentation technologies like CRISPR-engineered strains of *F. venenatum* are predicted to reduce the potential for global warming by an additional 4%–61% (19). All things considered, mycelium stands out as a natural fortificant and an eco-friendly protein source that effectively addresses protein and micronutrient deficiencies and sustainability concerns without the environmental and chemical costs associated with conventional fortification techniques.

## 7 Challenges and future perspectives

Despite its enormous potential, good nutritional profile, and sustainable production, mycelium still faces several challenges that limit its widespread adoption as a food fortificant. Consumer acceptance is a significant hurdle, as people assume mycelium is mold, thus lowering their willingness to buy, despite its benefits (107). Food neophobia, a lack of awareness of Mycelium’s clean-label credentials, and limited experience with substances derived from fermentation further impede market adoption. Coordinated efforts are needed to develop transparent labeling, customer education, and product format optimization to include mycelium in food products. Sensory quality and palatability are directly linked to consumer acceptance. Mycelium has a natural advantage for developing meat-alternative products due to its rich umami aroma; however, using it in sweet products is a challenge. A study by Stoffel et al. (29) on incorporating *P. albidus* mycoprotein into chocolate cookies found that it contributed to the texture and sensory characteristics of the cookies. Conversely, SSF-derived *R. oligosporus* biomass has been found to yield better sensory and textural results than SmF-derived biomass (71). These findings show that different fungus species, fermentation techniques, and inclusion levels result in quite different sensory consequences. Enhancing palatability and increasing consumer acceptance of mycelium-based functional foods will require optimizing formulation techniques, such as aroma and taste masking, enzymatic treatment, or mixing with other ingredients.

Allergenicity is another important aspect that needs to be considered for food, as it may limit its wider use. Some consumers have experienced gastrointestinal issues from the fungal mycoprotein *F. venenatum*, without true immune (IgE) allergy (108). In rare cases, certain cross-reactive allergens, such as the 60S acidic ribosomal protein P2, have been identified as triggers of genuine IgE-mediated hypersensitivity (109). Even though mycoprotein allergies are rare, it is important to note that allergen testing, labeling, and detailed targeted allergen information should be provided when mycelium is introduced into food products.

More recent fungal species, such as *N. crassa* and *A. oryzae*, require comprehensive toxicological testing before regulatory authorization can be granted. On the other hand, there are 40 years of safety data for well-known microbes such as *F. venenatum* (16). The European Union has rigorous quality checks for novel foods, which are time-consuming and expensive, thereby preventing new mycelium species from entering the market. Another important challenge is maintaining consistent biomass and composition, which is very difficult to achieve during upscaling or across different bioreactor sizes, particularly in SSF, where upscaling is hindered by heterogeneous heat and mass transfer (17).

Submerged fermentation has been used in commercial *F. venenatum* production for 40 years, demonstrating its suitability for industrial-scale mycelial biomass production. It is preferred over SSF at the commercial level due to its precise control of pH, dissolved oxygen, nutrient levels in the media, and temperature, resulting in consistent quality, repeatable results, and a low contamination risk due to the closed system. In comparison, SSF has several advantages over SmF, such as lower capital and operating costs, lower energy and water use, and, most importantly, the utilization of agro-waste residues into high-value product ingredients, demonstrating sustainability and a circular economy. However, achieving consistent, repeatable results with SSF is difficult due to substrate variability and limited process control.

There is a crucial gap in understanding micronutrient bioavailability due to the lack of human clinical evidence. This is because animal models and *in vitro* bioaccessibility data have not yet been sufficiently translated into controlled human feeding trials with isotope-based biomarker validation (8).

Future studies and technical advancements across related fields will be essential to realizing mycelium’s full potential as a platform for sustainable food fortification. Novel technologies, such as precision fermentation, AI, and IoT-based fermentation and optimization, as well as the use of CRISPR and metabolic engineering, can offer a promising approach to simultaneously enhance mycelium’s ability to accumulate micronutrients, improve protein quality, and increase production efficiency while reducing the environmental impact (19, 20, 110). To determine the true bioavailability of important micronutrients, especially organically bound zinc and selenium accumulated through biofortification strategies, systematic human intervention trials employing isotope-dilution methodologies should be given priority. This will provide the clinical evidence base required to support regulatory health claims for mycelium-based fortified foods. In addition to being a cost-cutting measure, substrate engineering utilizing circular economy-aligned agro-industrial residues should be further investigated as a dual-function strategy in which waste streams concurrently function as sources of carbon and nitrogen as well as natural mineral carriers that mycelium can biotransform into bioavailable micronutrient pools (17, 18). In order to transform mycelium from a premium specialty ingredient into a widely available, population-level food fortification tool that can simultaneously address protein malnutrition and hidden hunger within a single, scalable biological platform, it is still necessary to achieve techno-economic price parity with conventional synthetic fortification ingredients through process intensification, strain improvement, and co-product valorization, as modeled by Risner et al. (111).

## 8 Conclusion

Mycelium has become a versatile and promising food ingredient, surpassing its role as just a protein source. This study demonstrates that mycelium provides high-quality protein in a single biological matrix, along with naturally occurring micronutrients, dietary fiber, and a variety of bioactive compounds, a combination that synthetic fortification cannot provide in a clean-label, ecologically sustainable manner. However, this compositional richness should be considered alongside digestibility and bioaccessibility, as fungal cell wall components such as chitin and β-glucans can hinder both. From a nutritional standpoint, mycelium offers a high mineral content, a robust amino acid profile, and the capacity to spontaneously synthesize vitamins such as ergothioneine and vitamin D_2_. Biofortification techniques, including zinc and selenium supplementation, UV exposure, encapsulated mineral delivery, and substrate alteration, can enhance these qualities. Consequently, mycelium serves as a flexible, programmable platform for food fortification that can address multiple micronutrient deficiencies. Large-scale mycelium production using a variety of substrates, including agro-industrial waste, is enabled by the adaptability of fermentation-based methods, such as submerged, solid-state, and precision fermentation. SmF has been industrially proven for over 4 decades to produce biomass with consistent quality. In contrast, SSF offers low cost and energy with the circular economy by utilizing agro-waste and is better suited for smaller-scale production. Compared to conventional animal protein and chemically produced food additives, this method lessens environmental impact and upholds the ideals of the circular economy. Its potential as a mainstream ingredient is further demonstrated by its effective use across a variety of food categories, including meat substitutes, baked products, dairy substitutes, drinks, and nutraceuticals. Sensory and textural properties differ by fermentation method, species cultivated, the nutrients in the media used, and their levels of incorporation into food products, underscoring the need for formulation and optimization that consider consumer acceptance. Coordinated advancements in consumer education, regional regulatory harmonization, fermentation technology scaling, and human clinical studies to verify the bioavailability of mycelium-derived micronutrients in actual diets are necessary to realize this full potential. Mycelium may be positioned as a clean-label, sustainable, and scientifically verified solution to address hidden hunger, protein deficiencies, and the environmental limitations of the current global food fortification strategy with further research and funding.
