# Supplementary material for: Mycelium as a natural food fortificant: production, micronutrient enrichment, processing, and application in functional foods
Source: Front Nutr. 2026 Aug 13;13:1890681. doi: 10.3389/fnut.2026.1890681 (PMC13520741; doi:10.3389/fnut.2026.1890681)
Supplement: Supplementary file 1 [file Supplementary_file_1.docx]

**Mycelium as a Natural Food Fortificant: Production, Micronutrient Enrichment, Processing, and Application in Functional Foods**

**Declaration**

An AI tool was used solely for Figure 5, and the authors carried out all scientific content, interpretation, and validation.

**Prompt**

Strategies for micronutrient enrichment in mycelium encompass multiple complementary approaches aimed at improving the nutritional density and bioavailability of fungal biomass. Natural accumulation mechanisms exploit the inherent capacity of mycelia to uptake and biotransform minerals such as iron, zinc, selenium, and calcium from growth substrates into bioaccessible organic forms. Biofortification approaches further enhance this process through substrate supplementation, metabolic regulation, and controlled cultivation strategies, while vitamin enhancement strategies focus on stimulating the biosynthesis or exogenous incorporation of vitamins such as B-complex and ergocalciferol (vitamin D₂). In addition, encapsulation-assisted enrichment has emerged as an advanced strategy to protect sensitive micronutrients, improve retention during processing, and enable targeted delivery within fortified mycelial matrices. Develop an image for this.

**Result**

The image I provided with the manuscript

**AI Tool Details**

Generative AI technology: OpenAI Image Generation

Model: GPT-Image-1 (ChatGPT Image Generation Model)

Version: GPT-Image-1 (OpenAI does not publicly provide version numbers such as v1.2 or v2)

Developer/Source: OpenAI

Platform: ChatGPT ([https://chatgpt.com](https://chatgpt.com/))
